# Handheld tunable focus confocal microscope utilizing a double-clad fiber coupler for *in vivo* imaging of oral epithelium

**DOI:** 10.1117/1.JBO.22.5.056008

**Published:** 2017-05-25

**Authors:** Cory Olsovsky, Taylor Hinsdale, Rodrigo Cuenca, Yi-Shing Lisa Cheng, John M. Wright, Terry D. Rees, Javier A. Jo, Kristen C. Maitland

**Affiliations:** a Texas A&M University, Biomedical Engineering Department, College Station, Texas, United States; b Texas A&M University College of Dentistry, Department of Diagnostic Sciences, Dallas, Texas, United States; c Texas A&M University College of Dentistry, Department of Periodontics, Dallas, Texas, United States

**Keywords:** confocal microscopy, microendoscopy, endomicroscopy, *in vivo* imaging, cancer

## Abstract

A reflectance confocal endomicroscope with double-clad fiber coupler and electrically tunable focus lens is applied to imaging of the oral mucosa. The instrument is designed to be lightweight and robust for clinical use. The tunable lens allows axial scanning through >250  μm in the epithelium when the probe tip is placed in contact with tissue. Images are acquired at 6.6 frames per second with a field of view diameter up to 850  μm. *In vivo* imaging of a wide range of normal sites in the oral cavity demonstrates the accessibility of the handheld probe. *In vivo* imaging of clinical lesions diagnosed as inflammation and dysplasia illustrates the ability of reflectance confocal endomicroscopy to image cellular changes associated with pathology.

## 1 Introduction

Reflectance confocal microscopy provides three-dimensional (3-D) images of cellular and epithelial morphology to enable early cancer detection in a number of organs.^1^ Contrast in reflectance confocal imaging is generated by differences in refractive index of cellular components.^2^ In amelanotic epithelial tissue, increases in nuclear refractive index, DNA content, and chromatin texture result in increased light scattering with progression of neoplasia.^3^^,^^4^ Depending on tissue type, malignancy, and scattering coefficient of epithelium, imaging can be achieved partially or entirely through the epithelial layers using near-infrared light.^5^^,^^6^

While application in the oral cavity has been limited, reflectance confocal microscopy has been developed and validated as a noninvasive imaging tool in dermatology to characterize skin tissue,^7^^,^^8^ detect and diagnose malignancy,^9^ assess tumor margins, and monitor treatment.^10^ A limited number of publications have reported progress of reflectance confocal imaging of normal oral mucosa^11^^–^^15^ and clinical imaging of oral pathologic conditions.^16^^–^^19^ A recent review indicates the potential clinical role for *in vivo* reflectance confocal microscopy in stomatology given the correspondence of confocal images of oral tissue with histology images.^20^ Technical challenges that need to be addressed to apply reflectance confocal microscopy more broadly in the oral cavity include increased accessibility of all sites in the oral cavity; reduced motion artifacts; and reduced size, weight, and rigidity of probes.

Oral precancerous lesions and conditions, such as leukoplakia/erythroplakia and lichen planus, can be heterogeneous or diffuse and present with protean clinical features.^21^ Lesions in the oral mucosa have characteristic histologic features that allow them to be classified as malignancy, dysplasia, or other pathologic conditions. We have reported on a handheld confocal endomicroscope designed to acquire images of oral epithelium *in vivo* to characterize cellular and tissue morphology, akin to histology.^15^ This technology has the potential to improve the clinical assessment of oral pathologic conditions by providing noninvasive microscopic surveillance of lesions *in vivo*. While histology of oral mucosa uses a cross-section cut of fixed biopsy tissue to analyze morphology of the epithelium and lamina propria in a single tissue section, confocal microscopy can now be applied *in vivo*, generating *en face* images of cell and tissue morphology throughout the epithelium.

The traditional stage-scanning of benchtop confocal microscopes to generate images of multiple depths in a specimen is impractical *in vivo*. Axial scanning has been realized using several alternative techniques amenable to *in vivo* imaging, including mechanical scanning,^22^^,^^23^ depth-to-wavelength encoding,^24^ and tunable focal length elements.^25^^–^^27^ The endomicroscope presented here employs an electrically tunable lens (ETL) to change the focus of the microscope while it is in contact with tissue. The ETL axial scanning mechanism was selected for its capability to tune the focal plane in the sample without significant mechanical motion, while achieving a focal length range suitable for the epithelial depth of interest.

Practical design is needed for imaging oral epithelium with confocal endomicroscopy to determine efficacy of optical biopsy results,^18^ including design for reliability and ergonomics. Building on our previously reported confocal endomicroscope,^15^ we have made several enhancements toward a more rugged and user-friendly device appropriate for clinical use. First, a double-clad fiber coupler replaces the separate illumination and detection optical fibers. The double-clad fiber delivers light through the single-mode core to the optics in the handheld probe and collects the reflectance signal through the inner cladding in a partially coherent detection scheme. The faces of the core and inner cladding inherently lie in the same plane greatly simplifying alignment, which is especially important in a handheld device with such strict requirements on the alignment of a confocal pinhole for preservation of high resolution. Separating the illumination and detection paths in the fiber coupler also allows removal of a beamsplitter and associated mount from the handheld unit, reducing size and weight to minimize motion artifacts. The custom objective lens^15^ has also been modified to optimize the imaging range in epithelial tissue. A coverslip cemented to the lens tip shifts the short working distances to a range with better image quality in exchange for reducing the range by 100  μm. Finally, the handheld device housing has been replaced with a new 3-D printed housing to reduce the weight by 1.14 pounds and the size by ∼10% in each dimension.

Using this enhanced handheld confocal endomicroscope with a double-clad fiber coupler and ETL axial scanning, we have acquired images of oral epithelium *in vivo*. Images are shown for a wide range of normal oral mucosa sites, including the difficult to reach retromolar trigone. Furthermore, images of clinical lesions are presented to demonstrate microscopic changes associated with pathology.

## 2 Methods

The reflectance confocal microscope consists of a handheld rigid probe housing the optics and scanning unit, and an equipment cart containing the laser, detector, and control and acquisition electronics. The probe is tethered to the cart with a black flexible corrugated wire loom that routes the optical fiber and the control wires for the mirrors and ETL. A fiber-coupled 811-nm diode-pumped solid-state laser (Crystalaser, DL808-120-O) is used as the source for reflectance imaging. The laser’s single-mode (SM) fiber is spliced to the 4-μm diameter core of the double-clad fiber, delivering coherent illumination to the handheld probe.

**Fig. 1:**
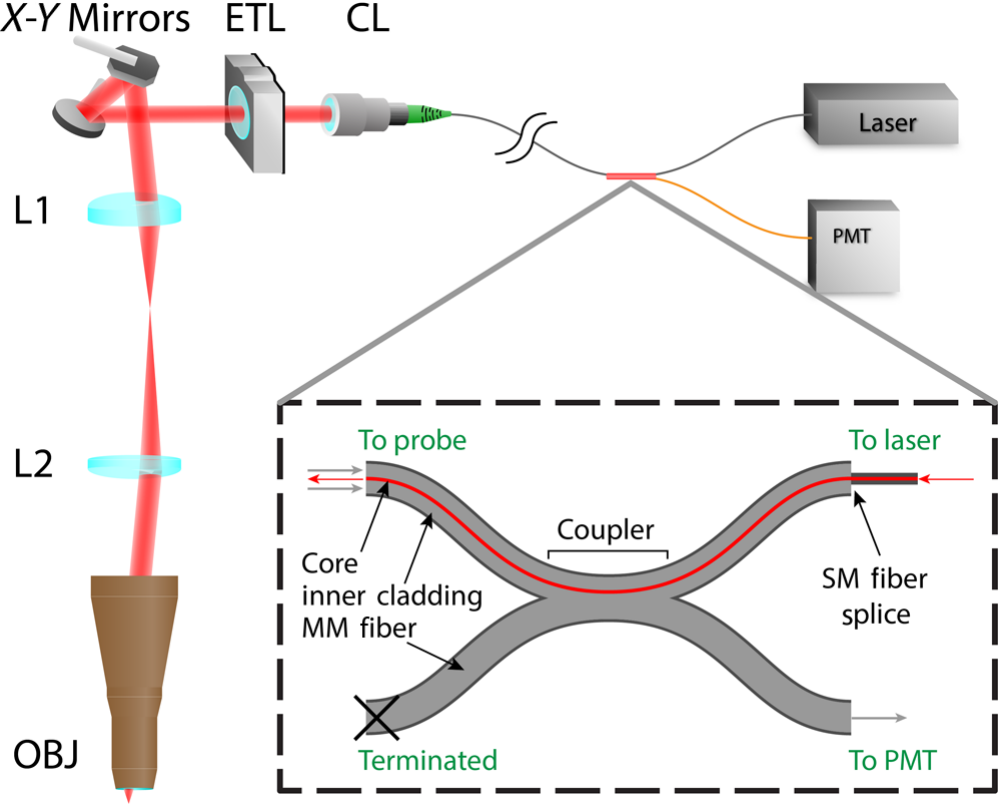
Schematic of handheld confocal endomicroscope. Probe components: collimator lens, ETL, X−Y scanning mirrors, 25-mm (L1) and 50-mm (L2) relay lenses, and custom objective (OBJ). (Inset) The laser is coupled with an SM fiber to the core of the double-clad fiber. Reflected light is collected by the inner cladding, passed through the double-clad fiber coupler to the MMfiber, and detected by a PMT.

In the probe (Fig. 1), the output of the fiber is collimated before passing through the tunable lens. The ETL (Optotune, EL-6-18) is a deformable polymer surface in a lens package that allows tunability of the divergence of the beam and, ultimately, the working distance of the system. The ETL is positioned to optimize the working distance range within the sample, while avoiding back-reflections from lens surfaces within the system and minimizing magnification change with scanning. The two-dimensional (2-D) beam scanning unit (Cambridge Technology, CRS 8 kHz resonant scanner and 6200 H galvanometer scanner) consists of a resonant scanning mirror with a sinusoidal line scan at a frequency of 7.91 kHz and a galvanometer scanning mirror with a smoothed sawtooth scan at a frequency of 6.6 Hz, the frame rate. The beam is relayed to the objective lens with a 2× relay made from two custom achromatic doublets (manufactured by Optics Technology, Inc.) that are designed to balance aberration for off-axis beams. The scan angles of the mirrors are set to achieve a field of view diameter up to 850  μm.

The ETL is placed before the scanning mirrors, so the chief rays are not altered when tuning the focus. For an ideal telecentric imaging relay, this means that magnification is constant over the axial scanning range. Another valid position for the ETL is in the back focal plane of the objective, where all chief rays pass through the center of the aperture. However, the ETL is large and would be too obstructive for an intraoral probe. Since the ETL is in front of the relay lens (Fig. 1), the available focal range will be reduced due to longitudinal magnification of the relay and objective lenses. Even so, the focal range for this configuration is acceptable. An additional benefit to placing the ETL before the scanning module is that aberrations due to off axis rays entering the ETL are avoided.

A coverslip glass (Schott Nexterion Glass D) polished to a nominal thickness of 150 μm and coated for water immersion was cemented to the last surface of the custom 0.7 numerical aperture, 5-mm focal length water dipping objective previously reported^15^ to shift the nominal working distance closer to the glass surface. The modified objective focuses the beam at a nominal working distance of 180  μm from the coverslip surface. In effect, this lens configuration is optimized for more superficial imaging in comparison to the previous design that had a nominal working distance of 305  μm.^15^ The working distance can be adjusted between 0 and 265  μm by supplying a current between 135 and 0 mA, respectively, to the ETL. The tip of the objective is 6.5 mm in diameter to enable localization of the imaging field within clinical lesions.

**Fig. 2:**
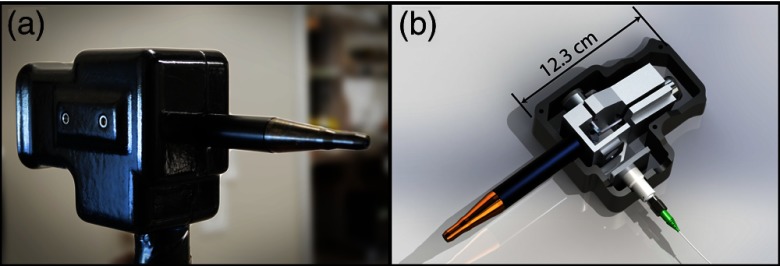
Handheld reflectance confocal microendoscope. (a) Photograph of probe with plastic shell. (b) Solidworks rendering of internal components.

The components in the handheld probe are mounted on a machined aluminum body and housed by a 3-D printed acrylonitrile butadiene styrene plastic shell (Fig. 2). The shell has been washed with acetone to temporarily dissolve the surface allowing it to smooth for a nicer appearance, better grip, and effective cleaning.

The 2×2 coupler is a double-clad fiber with the inner cladding coupled to a multimode (MM) fiber (Castor Optics). The illumination light is delivered through the core to the probe, and the back reflected light is collected by the inner cladding. The double-clad fiber allows the use of a partially coherent detection scheme using a single fiber and common illumination and detection path.^28^ Since the core and inner cladding inherently lie in the same plane, the illumination and detection planes are automatically aligned, removing the need for frequent alignment. The 2×2 double-clad fiber coupler is designed for an inner cladding to core ratio of 5, the optimum for balancing resolution, speckle reduction, and signal collection.^29^ The theoretical axial resolution is 5.7  μm using the calculation for a partially coherent detector.^30^

The collected signal is transferred into the MM fiber by the coupler, and then detected by a photomultiplier tube (PMT, Hamamatsu, H9305-03). The signal from the PMT is amplified with a high bandwidth current-to-voltage amplifier (Hamamatsu, C9999) and digitized by an oscilloscope (National Instruments, PXI-N5122). Horizontal synchronization is achieved by using the sync signal from the control board of the resonant scanning mirror as the oscilloscope trigger. The vertical synchronization is software timed so that the galvanometer mirror scan and the image frame capture are started together. The program corrects the image for distortion from the nonlinear (sine wave) scan of the resonant scanning mirror by resampling the horizontal line with an inverse sine sampling interval.

The imaging system was used to collect images *in vivo* from the oral mucosa of healthy volunteers and of patients presenting with oral lesions in the Stomatology Clinic at the Texas A&M University College of Dentistry. All imaging was performed after protocol approval by the Texas A&M University College of Dentistry, Institutional Review Board, and informed consent by the study participant. Prior to imaging, a gauze pad soaked in 5% acetic acid solution is applied gently to the lesion for 1 min to enhance nuclear contrast.^31^ The objective lens of the confocal probe is placed in contact with the mucosa. Images can be acquired at a fixed imaging depth while the probe is translated over an area of tissue, or the probe can be positioned at a specific point of interest, such as on a lesion, and a full range axial scan can be acquired in five seconds, driven by the ETL. After clinical imaging of suspicious lesions, the same procedure of acetic acid application and imaging are performed on a contralateral, clinically normal tissue site. The patient then undergoes a biopsy procedure of the lesion site, which is processed for histopathology for diagnostic purposes as standard of care.

## 3 Results and Discussion

The axial response was measured by moving a mirror on a translation stage axially through the focus of the microscope. This was repeated at regular intervals of working distance by changing the ETL current. The full-width half-maximum (FWHM) of the axial response varies over the focal range due to aberration differences, ranging between 6  μm at the nominal working distance of the objective lens and 12  μm near the surface of the coverslip. Although the objective is well corrected for spherical aberration, aberration from the ETL and the coverslip may worsen resolution. At shorter working distances, there is also slight underfilling of the objective. The lenses are nominally designed for infinite conjugates so additional aberration will appear when the ETL changes the divergence. The probe is placed in contact with the tissue sample, and the depth of the image inside the tissue can be approximated using a calibration curve to convert ETL current to depth in μm. The calibration is obtained by decreasing the ETL current incrementally while using the mirror mounted on a linear stage to determine the focus using the position of maximum backreflected light. The >250-μm depth scan range is sufficient to image the epithelium of most sites in the oral cavity. The primary limitation is loss of signal collection or resolution when imaging through turbid tissue.

**Fig. 3:**
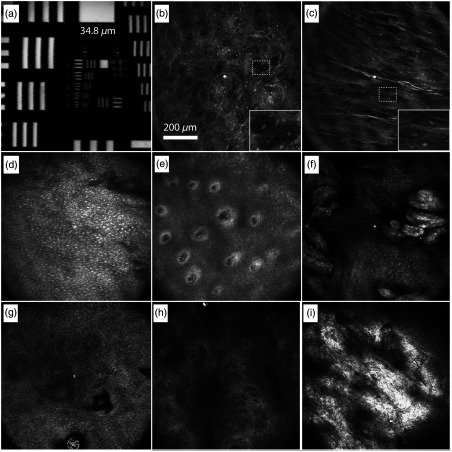
Confocal endomicroscope images of test target and normal oral mucosa *in vivo*. (a) 6× zoomed in and cropped image of 1951 USAF resolution test chart. Superficial layers (∼30-μm deep) of (b) buccal mucosa and (c) retromolar trigone. Insets show 3× zoom-in of individual cells. (d) Spinous layer ∼20-μm deep and (e) epithelial junction ∼100-μm deep in lateral tongue. (f) Lingual papillae on the surface of the dorsal tongue imaged ∼50  μm from the glass surface. (g) Superficial (∼35  μm) and (h) deeper (∼120  μm) layers in the gingival epithelium. (i) Lamina propria of labial mucosa ∼230-μm deep with blood vessels identified by arrows. Scale: (a) Width of the block feature at the top of the image is 34.8  μm. (b)–(i) 200  μm scale bar in (b) representative of scale in all tissue images.

A 1951 U.S. Air Force (USAF) resolution test target was imaged to evaluate image quality. Figure 3(a) shows a zoomed in and cropped image of group 9 to demonstrate image quality near the nominal working distance of the objective. The 0.85-μm sampling interval limits the resolution to the Nyquist frequency (in the x or y dimension) of 588 lines per mm (1.7-μm period). The measured lateral resolution using the FWHM of the derivative of the edge response is 1.4  μm.

Figures 3(b)–3(i) show *in vivo* imaging data from a wide range of oral mucosa sites in healthy volunteers to illustrate the capability and accessibility of the probe for clinical use. The endomicroscope can be used to access several hard to reach areas of the oral cavity, including the retromolar trigone behind the wisdom teeth. Images are shown from the superficial layers of the buccal mucosa of the cheek [Fig. 3(b), ∼30-μm deep], the retromolar trigone [Fig. 3(c), ∼30-μm deep], the lateral tongue [Fig. 3(d), ∼20-μm deep], and the vestibular gingiva [Fig. 3(g), ∼35-μm deep]. Stratified squamous epithelial cells of the granular layer of the retromolar trigone in Fig. 3(c) show an elongated shape compared to the circular shape of the cells near the surface of the buccal mucosa in Fig. 3(b), possibly due to stretching of the epithelium when the buccal mucosa is pulled out to allow imaging in the posterior oral cavity. The image at a similar depth in the lateral tongue in Fig. 3(d) is likely in the spinous cell layer of the epithelium with larger, brighter, and closely packed nuclei. The image captured around 100  μm in the lateral tongue, Fig. 3(e), shows the connective tissue papillae extending into the epithelium as evidenced by the bright rings of basal cells surrounding central cross sections of lamina propria. The image of the dorsal surface of the tongue, Fig. 3(f), shows epithelium of lingual papillae ∼50  μm from the surface of the last optical element. Images of the gingival epithelium, Figs. 3(g)–3(h), did not show bright nuclei but did show cell borders in the superficial layers (∼35-μm deep) and connective tissue papillae around 120-μm deep. Blood vessels, identified by arrows, can be seen in the lamina propria of the labial mucosa at a depth of ∼230  μm [Fig. 3(i)].

**Fig. 4:**
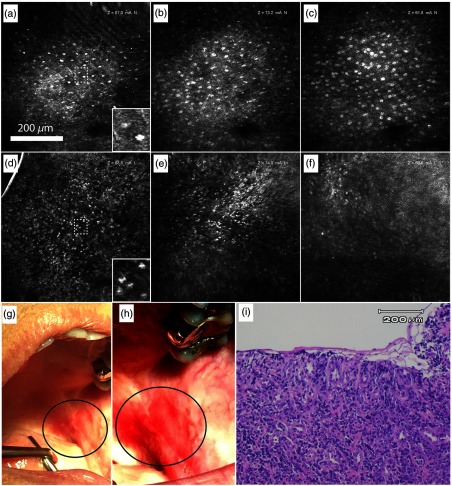
*In vivo* confocal endomicroscope images of clinically normal buccal mucosa at approximately (a) 75  μm, (b) 100  μm, and (c) 150  μm, and ulcerated buccal mucosa at (d) 75  μm, (e) 100  μm, and (f) 150  μm. Insets: 3× zoom-in of individual cells exhibit example nuclear size, shape, and spacing. (g) and (h) Clinical photographs with the ulcer circled. (i) H&E stained histology section of lesion showing complete loss of epithelium, diagnosed as ulceration with chronically inflamed granulation tissue.

Figure 4 shows confocal endomicroscope images captured *in vivo* from the posterior buccal mucosa of a patient in the Stomatology Clinic being evaluated for an ulcer. An ulcer is a localized defect in the surface of mucosa or skin.^32^ Images of a clinically normal contralateral site [Figs. 4(a)–4(c)] and the lesion [Figs. 4(d)–4(f)] are shown at depths of ∼75, 100, and 150  μm into the epithelium. The bright cell nuclei are well defined and some cell borders can be seen. The normal tissue shows nuclei ∼8- to 10-μm in diameter and increasing in density with depth. In the images of the lesion, the inflammatory cells in the granulation tissue in the ulcerated area have slightly smaller nuclei but are densely crowded together even in the relatively shallow 75-μm depth range [Fig. 4(d)]. Inflammatory cells found in an ulcer are primarily neutrophils in the fibrin layer and lymphocytes or plasma cells in the granulation tissue. The abundant dark staining of nuclei in these leukocytes indicates condensed chromatin in the nucleus, which would be expected to result in a bright reflectance signal in confocal microscopy. Lymphocyte nuclei are spherical in shape with a mean nuclear diameter of ∼4.5  μm.^33^ The nuclei of neutrophils are polymorphic and multilobular with three to five lobes.^34^ Although neutrophils (7.0-μm mean cell diameter) measure slightly larger than lymphocytes (6.2-μm mean cell diameter), the ratio of nuclear to cell volume of neutrophils (21%) is about half that of lymphocytes (44%).^33^ While the neutrophil nuclear perimeter may be comparable to lymphocyte nuclear circumference, the individual nuclear lobes in neutrophils are typically 2.5  μm or less in diameter. In Fig. 4(d), at 75-μm depth, several nuclei appear to have multilobular morphology.

The deepest image shown for the granulation tissue [Fig. 4(f)] is degraded likely due to the increased scattering from the nuclei dense morphology of the superficial tissue. Figures 4(g)–4(h) show clinical photographs of the imaged ulcer. The hematoxylin and eosin (H&E) stained histological section [Fig. 4(i)] of the biopsy obtained from the imaging site (ulcer) shows the disruption of the full thickness of the epithelium, leaving a fibrin layer and the underlying granulation tissue. The histopathological diagnosis was nonspecific ulceration with chronically inflamed granulation tissue, with no evidence of neoplasm. The features in the confocal images, including the lack of epithelial cells and the presence of small crowded nuclei in an unorganized structure, correspond well with histology.

**Fig. 5:**
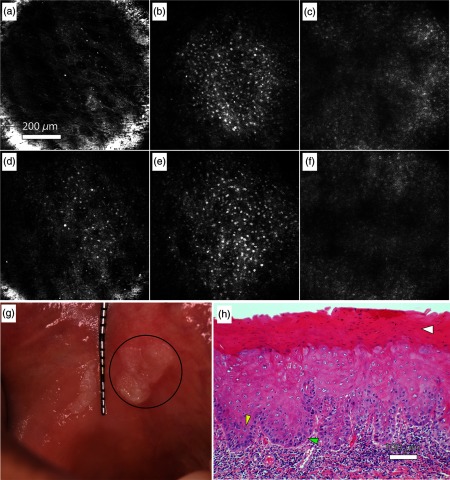
*In vivo* confocal endomicroscope images of clinically normal buccal mucosa at approximately (a) 10  μm, (b) 70  μm, and (c) 120  μm, and of buccal mucosa clinically diagnosed as leukoplakia at (d) 10  μm, (e) 70  μm, and (f) 120  μm. (g) Clinical photograph of the leukoplakia with the imaged lesion circled. (h) H&E stained histology section of lesion diagnosed as mild to focally moderate epithelial dysplasia. Yellow arrow: increased nuclear to cytoplasmic ratio. Green arrow: loss of polarity of basal cells. White arrow: hyperkeratosis.

Figure 5 shows endomicroscope images captured *in vivo* in a patient presenting with leukoplakia in the buccal mucosa. Images from the clinically normal contralateral site [Figs. 5(a)–5(c)] and the clinically white lesion [Figs. 5(d)–5(f)] are shown. Figure 5(g) shows a clinical photograph of the leukoplakia. Figure 5(h) shows an H&E stained histology section of the biopsy obtained from the imaging site within the lesion that was diagnosed as moderate epithelial dysplasia. While the nuclei in the confocal images of the lesion appear more similar through the depth scan, the images of the normal site illustrate the characteristic layered structure of normal stratified squamous epithelium. This difference may indicate irregular epithelial stratification in the dysplastic lesion. Figure 5(a) located near the surface of the epithelium of the clinically normal site shows a superficial layer of flattened cells with small nuclei, similar to the superficial epithelium of the buccal mucosa of a normal volunteer [Fig. 3(b)]. In Fig. 5(b), the slightly curved focal plane transitions at the outer rim of the field of view from the superficial layer to the spinous cell layer of the epithelium with larger, more closely packed nuclei than the superficial layer. Approaching the basement membrane, the cell nuclei have a dense arrangement in Fig. 5(c). In contrast, both Figs. 5(d) and 5(e) at 10 and 70  μm below the surface, respectively, show larger epithelial nuclei similar to nuclei in the spinous cell layer in the central area of Fig. 5(b). If the spinous cells at similar depths in the central areas of Figs. 5(b) and 5(e) are compared, the mean intensity of nuclei and the mean intensity of the cellular area as a whole are both increased in the lesion site. Figure 5(f) near the basement membrane appears similar to Fig. 5(c). The image [Fig. 5(h)] of the H&E stained histology slide obtained from the imaging site in the lesion was diagnosed as mild to focally moderate epithelial dysplasia. Histologic examination reveals keratinized mucosa with epithelial cytologic atypia. The thickened parakeratin layer (hyperkeratosis) characteristic of leukoplakia is identified in the H&E section by a white arrow. Altered cellular maturation is evident in roughly the lower half of the epithelium where the cells show varying degrees of both pleomorphism and nuclear hyperchromatism. The yellow arrow indicates a region of increased nuclear to cytoplasmic ratio, and the green arrow identifies an example of loss of polarity of basal cells. Although the image in Fig. 5(h) is focused on a small portion of the epithelium to show cellular detail, cytologic changes are confined to the epithelium throughout the histologic section, and there is no evidence of invasion into the lamina propria. Since the hyperchromatism in Fig. 5(h) may be attributable to increased nuclear chromatin content or higher DNA turnover in dysplastic epithelium, it is interesting that the reflectance signal from nuclei is greater in the lesion site [Fig. 5(e)], possibly due to excess chromatin.

## 4 Conclusion

In this paper, we present a robust handheld tunable reflectance confocal endomicroscope for imaging oral mucosa *in vivo*. We have designed this instrument to be maneuvered within the oral cavity to image sites that have a higher incidence of neoplasia. The ETL allows rapid axial scanning of the focal plane through the epithelium, without mechanical scanning, when the probe tip is placed in contact with the tissue. A double-clad fiber coupler is used to simplify optical alignment, optimize speckle reduction, and reduce overall housing size. Confocal images are shown for a range of sites and at various depths in the oral cavity of a normal volunteer. Additionally, clinical imaging of two stomatology patients demonstrates the ability of the confocal endomicroscope to resolve nuclear changes associated with inflammation and dysplasia.

Handheld confocal microscopy has the potential to resolve a subset of the diverse set of histological features associated with dysplasia and cancer. Reflectance confocal images appear quite different from conventional histology sections, but modifications can be made to image acquisition or presentation to emulate histology. Digital staining of reflectance confocal images has been applied in dermatology to mimic H&E staining in histopathology.^35^ The small field of view of confocal microscopy can be alleviated with image or video mosaics^36^^,^^37^ by fixing the focal depth of the microscope using the tunable lens and scanning laterally over a large area with the handheld probe. 3-D acquisition of confocal images allows image presentation in 3-D or 2-D *en face* or cross section. *En face* 2-D images are typically presented because lateral resolution is superior to axial resolution in confocal microscopy. In handheld confocal imaging, motion artifacts may affect the ability to generate 3-D images. To achieve high-fidelity cross-section images, the tunable lens (axial scan) scanning speed can be set to serve as the frame scan in image acquisition.^26^ Automated image processing and classification may provide real-time feedback to clinicians.^38^^–^^40^ Although near-infrared light enables light penetration through the epithelium, imaging resolution degrades with depth due to tissue scattering, which may limit the extent of disease classification.

Images from two patients are presented here; further data acquisition is necessary to evaluate the capability of reflectance confocal microscopy to differentiate among (pre)malignant, benign, and normal oral mucosa *in vivo*. With further study, the technology may prove useful to guide tissue biopsy, improve diagnostic yield, or monitor treatment efficacy.
